# KPT-330, a potent and selective exportin-1 (XPO-1) inhibitor, shows antitumor effects modulating the expression of cyclin D1 and survivin in prostate cancer models

**DOI:** 10.1186/s12885-015-1936-z

**Published:** 2015-12-01

**Authors:** Giovanni Luca Gravina, Andrea Mancini, Patrizia Sanita, Flora Vitale, Francesco Marampon, Luca Ventura, Yosef Landesman, Dilara McCauley, Michael Kauffman, Sharon Shacham, Claudio Festuccia

**Affiliations:** 1 Department of Biotechnological and Applied Clinical Sciences, Laboratory of Radiobiology, University of L’Aquila, L’Aquila, Italy; 2 Pathology Division, San Salvatore Hospital, L’Aquila, Italy; 3 Karyopharm Therapeutics, Newton, MA USA

**Keywords:** Prostate cancer, Cyclin D1, Tumor suppressor proteins, CRM-1, XPO-1, KPT-330, Selinexor, Selective Inhibitors of Nuclear Export (SINE)

## Abstract

**Background and aims:**

Increased expression of Chromosome Region Maintenance (CRM-1)/exportin-1 (XPO-1) has been correlated with poor prognosis in several aggressive tumors, making it an interesting therapeutic target. Selective Inhibitor of Nuclear Export (SINE) compounds bind to XPO-1 and block its ability to export cargo proteins. Here, we investigated the effects of a new class of SINE compounds in models of prostate cancer.

**Material and methods:**

We evaluated the expression of XPO-1 in human prostate cancer tissues and cell lines. Next, six SINE (KPT-127, KPT-185, KPT-205, KPT-225, KPT-251 and KPT-330) compounds having different potency with broad-spectrum, tumor-selective cytotoxicity, tolerability and pharmacokinetic profiles were tested in a panel of prostate cancer cells representing distinct differentiation/progression states of disease and genotypes. Two SINE candidates for clinical trials (KPT-251 and KPT-330) were also tested in vivo in three cell models of aggressive prostate cancer engrafted in male nude mice.

**Results and conclusions:**

XPO-1 is overexpressed in prostate cancer compared to normal or hyperplastic tissues. Increased XPO-1 expression, mainly in the nuclear compartment, was associated with increased Gleason score and bone metastatic potential supporting the use of SINEs in advanced prostate cancer. SINE compounds inhibited proliferation and promoted apoptosis of tumor cells, but did not affect immortalized non-transformed prostate epithelial cells. Nuclei from SINE treated cells showed increased protein localization of XPO-1, survivin and cyclin D1 followed by degradation of these proteins leading to cell cycle arrest and apoptosis. Oral administration of KPT-251 and KPT-330 in PC3, DU145 and 22rv1 tumor-bearing nude mice reduced tumor cell proliferation, angiogenesis and induced apoptosis. Our results provide supportive evidence for the therapeutic use of SINE compounds in advanced/castration resistant prostate cancers and warrants further clinical investigation.

## Background

Prostate cancer (PCa) is the second leading cause of cancer mortality in males >40 years of age in the USA and the third most common cause of cancer-related mortality in males [1]. PCa is generally a slow developing cancer, and 5- and 10-year relative survival rates of early stage PCa are 99 and 95 %, respectively [2]. Although hormone therapy is initially very effective, almost all tumors relapse to a hormone refractory stage. In the past, it was presumed that the expression of the androgen receptor (AR) is lost in the cells of advanced, hormone-refractory tumors but AR is rarely lost in human PCa specimens in vivo, even in those of CRPC [3]. Not only that AR is not lost, but it is transcriptionally active in the majority of recurrent CRPC [4]. There is experimental evidence that the Akt, mTOR and glycogen synthase kinase-3 (GSK-3β) pathways are involved in AR signaling [5, 6]. GSK-3β binds to the AR, forming a complex in the cytoplasm that are then imported into the nucleus upon androgenic stimulation. Inhibition of GSK-3β by activation of Akt/mTOR pathways results in increased nuclear export of AR and this export can be abrogated by the inhibition of XPO-1. GSK-3β/XPO-1 activity also regulates the levels of several nuclear and cytoplasmic proteins including survivin [7, 8] and cyclin D1 [8], which modulate cell division and apoptosis. Advanced castration resistant prostate cancer (CRPC) tumors are characterized by the activation of PI3K/AKT [9, 10]. One of the major effects of the activation of this pathway is XPO-1 dependent nuclear export of the tumor suppressor protein (TSP) FOXO into the nucleus, thus abolishing its activity [11]. Normally, low levels of FOXO protein are found in the cytoplasm. Shortly after SINE treatment, FOXO begins to accumulate in the nucleus where it binds to DNA and induces gene transcription that results in cancer cell death [12, 13].

Cancer cells utilize nuclear-cytoplasmic transport through the nuclear pore complex to effectively evade apoptosis and promote growth [14, 15]. XPO-1-mediated export is increased in various cancers [16–19]. Examples of nuclear proteins that are exported into the cytoplasm in cancer include the drug targets topoisomerase (topo) IIα [20] and tumor-suppressor proteins such as p53 [21], p21 [22], and p27 [23]. Use of XPO-1 inhibition in cancer therapy has been met with limited success. The first studied XPO-1 inhibitor was the anti-fungal natural antibiotic leptomycin B. It was found to efficiently inhibit nuclear export [24], but induced acute toxicities both in vitro [25] and in a human phase I trial [26]. Other XPO-1 inhibitors [for review see 14, 15] examined in various studies include compounds such as ratjadone [27], KOS-2464 [28], FOXO export inhibitors [29], valtrate [30], acetoxychavicol acetate [31], CBS9106 [32] and SINE (Selective Inhibitors of Nuclear Export) [33–43]. Recent publications have indicated that SINE compounds may be effective against various malignancies, including leukemia [34], breast cancer [35, 36] kidney cancer [37], mantle cell lymphoma [38], melanoma [39], multiple myeloma (MM) [40], pancreatic cancer [41], mesotelioma [42] and metastatic PCa [43]. For these reasons, we focused our attention of XPO-1 inhibition as therapeutic tool for the treatment of cancer, including PCa. In this study, we show that XPO-1 inhibition using SINE compounds: KPT-185, KPT-205, KPT-225, and KPT-127 reduced, in a panel of PCa cells, XPO-1-dependent nuclear export of different proteins including AR, Foxo3a, and survivin, modulating cell cycle progression through both a G1 and a G2/M-arrest followed by apoptosis. Clinical candidate KPT-251 and KPT-330 were also tested in vivo in three models of aggressive PCa.

## Methods

### Reagents and drug preparation

All materials for tissue culture were purchased from Hyclone (Cramlington, NE, USA). Plasticware was obtained from Nunc (Roskilde, Denmark). Antibodies including p-GSK3β Ser9 [sc11757], GSK3β [H76, sc-9166], XPO-1 [sc-5595], P53 [sc126], p-Akt Ser473 [sc-135651], p-Akt Thr308 [sc135650] and Rad-51 [sc8349], were purchased from Santa Cruz (SantaCruz, CA, USA). Antibodies including CD3 [ab28364], CD68 [ab955], FoxO3a [ab37409], Iκb [ab32518] were purchased from Abcam (Cambridge UK). An antibody against Cyclin D1 [#2878] was purchased from Cell Signaling (Danvers, MA, USA). A FAS antibody [VP-F702] was purchased from Vector labs (Burlingame, CA, USA). Ki67 antibody (clone MIB-1) was purchased from Dako (Dako Italia, Cernusco sul Naviglio [MI], Italy). Tunel assay kit [S71003] was purchased from Merck KGaA (Darmstadt, Germany). Survivin antibody was purchased from Biorbyt.

SINE compounds (KPT-127, −185, −205, −225, −251, −330) were provided by Karyopharm Therapeutics Inc., Natick, MA. KPT-251 and −330 are suitable for in vivo use. SINE compounds were dissolved in DMSO and stored at −20 °C until use.

### Human tissues

A cohort of 50 adult patients with clinically localized PCa, collected as previously described [7]. In addition, we analyzed 4 lymph nodal metastases from PCa patients and 57 samples of two tissue arrays from primary tumors (49 cases) and bone metastases (8 cases) purchased from US Biomax (Rockville, MD, USA). A total of 121 cases including 99 primary tumors, 4 lymph node metastases and 8 bone metastases were included. This research was carried out in accordance with the Helsinki Declaration and the study was approved by the San Salvatore Hospital Ethics Committee, L'Aquila, Italy with Deliberation n. 89/2006. We obtained also a written informed consent of patients.

### Immunohistochemistry

XPO-1 expression was evaluated on 4 μm tissue sections cut from blocks selected for the presence of representative tumor tissue. The pathologic evaluation and IHC results were interpreted by a uro-pathologist. First, nuclear and cytoplasmic staining of XPO-1 in tumor tissue was scored blindly (LV) using a semi-quantitative immunoreactivity scoring (IRS) system. Category A scored the intensity of immunostaining as 0 (no immunostaining), 1 (weak immunostaining), 2 (moderate immunostaining), and 3 (strong immunostaining). Category B scored the percentage of immunoreactive cells as 0 (none), 1 (<10 %), 2 (10–50 %) and 3 (>50 %). Multiplication of A and B resulted in an IRS from 0 to 9. An IRS of 4 or greater was considered high for expression of XPO-1. This analysis was performed for both nuclear and cytoplasmic staining. Global staining (GIRS) was the sum of nuclear and cytoplasmic staining and ranged between 0 and 18. A GIRS of 8 or greater was considered high for expression of XPO-1.

### Cell lines

Four commercial (LnCaP, 22rv1, DU145 and PC3) and twelve non-commercial (LAPC-4 [44], CWR22, PCb2 [45], PC3Lymphnode [45], PC3M, PC3M-pro4 [46] and PC3M-Ln4 [47], LnCaP-104S [47], LnCaP104R1 [47], LnCaP-C81 [47], C4-2B [48], VCAP [49] and DuCaP [49) cell lines were selected for the in vitro experimental studies for their biological characteristics: LAPC-4 (Androgen receptor [AR] positive, androgen dependent with low Akt/mTOR activities, p53 wt); LnCaP (AR positive, androgen dependent with high Akt/mTOR activities, p53 wt); LnCaP-C81 and LnCaP-C4-2B (AR positive, androgen independent with high Akt/mTOR activities, p53 wt); 22rv1 (AR positive, androgen independent with low Akt/mTOR activities, p53 wt); PC3 (AR negative, with high Akt/mTOR activities and no p53 function (p53 del) and DU145 (AR negative, with low Akt/mTOR activities and mutant p53). PC3 and DU145 have been also transfected with AR to obtain PC3AR [50] and DU-AR [51]. Benign prostatic hyperplasia line (BPH1), and Prostatic epithelial lines (EPN and RWPE-1) were used as non-neoplastic controls. Cells were routinely cultured in appropriate medium supplemented with 10 % fetal bovine serum (FBS), 1 % pen-strep and 1 % L-glutamine (Invitrogen Corporation, Carlsbad, CA). To minimize the risk to work with misidentified and/or contaminated cell lines, DNA profiling was periodically carried out in-house to authenticate cell cultures. DNA was isolated from cell lines using a standard DNA isolation kit. STR profiling was performed by using GenePrint® 10 System (Promega Corporation, Madison, WI). An eight-capillary 3500 Genetic Analyzer (Applied Biosystems Life Technologies Europe BV, Monza, Italy) was used to separate and identify alleles using standard procedures. GenePrint® 10 System allows co-amplification and detection of eight human loci required by the guidelines ASN-0002. For non-commercial cell lines, the authentication process was carried out by comparing STR-fingerprints with those published by Adri van Bokhoven and co-workers [52]. In addition, cell lines were stocked at very low passages and used at <15-20 subcultures.

### Growth assays

Cells were seeded at a density of 2 x 10^4^ cells/mL in 24-well plates. Cells were left to attach and grow in 5 % FCS DMEM for 24 h. After this time, cells were maintained in the appropriate culture conditions. Morphological controls were performed every day with an inverted phase-contrast photomicroscope (Nikon Diaphot, Tokyo, Japan) before cell trypsinization and counting. Cells were trypsinized and resuspended in 1.0 ml of saline, then counted using a NucleoCounter™ NC-100 (automated cell counter systems, Chemotec, Gydevang, Denmark). The effect on cell proliferation was measured by taking the mean cell number with respect to controls over time for the different treatment groups.

### Cell viability and apoptosis assay

Viable cells were counted by using the NucleoCounter™ NC-100 (automated cell counter systems, Chemotec, Cydevang, DK). Apoptosis was evaluated by using Tali® Apoptosis Kit - Annexin V Alexa Fluor® 488 & Propidium Iodide-based, (Life Technologies Italia, Monza, Italy). Stained cells were then measured on a Tali® Image-Based Cytometer. Apoptosis was further confirmed by FACS analysis following the instructions of the manufacturer.

### Western blot

Cytoplasmic and nuclear protein extracts were obtained by using the Nuclear/Cytosol Fractionation Kit from Biovision Inc. (Milpitas, CA, USA). Cell extracts and conditioned media from treated and untreated cells were electrophoresed under reducing conditions and transferred to nitrocellulose filter (Schleicher and Schuell GmbH, Dassel, Germany). Non specific binding sites were blocked for 1 h in 5 % non-fat dried milk in a Tris buffer containing 20 mM Tris and 137 mM NaCl (pH 7.6). Blots were incubated with 1 μg/ml of primary antibody diluted in blocking solution for 1 h at room temperature, washed and then incubated for 1 h in secondary antibody diluted 1:3000 in blocking solution. Following an additional wash, reactive bands were visualized by a chemiluminescent detection kit (Supersignal, Perbio Science, Tattenhall, UK) using Bio-Rad gel Doc™ (Bio-Rad Laboratories S.r.l., Milan, Italy).

### Xenograft model

Male CD1 nude mice (Charles River, Milan, Italy) were maintained under the guidelines established by the University of L’Aquila, Medical School and Science and Technology School Board Regulations. Experiments on animals have been approved by your local IRB in compliance with the Italian government regulation n.116 January 27, 1992 for the use of laboratory animals which is line with ARRIVE guidelines. All mice received subcutaneous flank injections of 1 x 10^6^ PC3, DU145 or 22v1 cells. Tumor growth was measured bi-weekly with a Vernier caliper (length x width). Tumor weight was calculated according to the formula: TW (mg) = tumor volume (mm^3^) = d2 x D/2, where d and D are the shortest and longest diameters, respectively. The effects of the treatments were examined as previously described [53]. Animals were sacrificed by carbon dioxide inhalation and tumors were subsequently removed surgically. A piece of tumor was frozen in liquid nitrogen for protein analysis and another piece was fixed in paraformaldehyde overnight for immunohistochemical analyses.

### Martius yellow-brilliant crystal scarlet blue technique

Stains were purchased from HD Supplies (Aylesbury, UK) and used to analyze the presence of red cells in tumor tissue and blood vessels, as well as to better evaluate the presence of micro-thrombi and bleeding zones. Martius yellow, a small molecule dye, together with phosphotungstic acid in alcoholic solution stains red cells. Early fibrin deposits may be colored, but the phosphotungstic acid blocks the staining of muscle, collagen and connective tissue fibers. Brilliant crystal scarlet, a medium sized molecule, stains muscle and mature fibrin. Phosphotungstic acid removes any red stain from the collagen. The large molecule dye aniline blue stains the collagen and old fibrin.

### Hemoglobin assay

Indirect evaluation of angiogenesis was performed by using tumor hemoglobin levels as previously described [53]. Tumors were homogenized in double-distilled water. Eighty microliters of homogenates were mixed with 1 ml of Drabkin’s solution and incubated for 15 min at room temperature. After centrifugation at 400 x g for 5 min, the supernatants were subjected to absorbance measurement at 540 nm. The absorption, which is proportional to hemoglobin concentration, was divided by tumor weight.

### Treatments

Mice were treated by oral gavage with either vehicle control (Pluronic F-68/PVP-K29/32), KPT330 or KPT251. Before tumor injection, animals were randomized into seven treatment groups as follows: Group 1: mice (10 animals) receiving 100 μl vehicle PO; Group 2: mice (10 animals) receiving 100 mg/kg KPT-251 every two days (Monday and Friday)/week PO; Group 3: mice (10 animals) receiving 30 mg/kg KPT-251 every two day/week PO; Group 4: mice (10 animals) receiving 10 mg/kg/day PO; Group 5: mice (10 animals) receiving 30 mg/kg KPT-330 every two days/week PO; Group 6: mice (10 animals) receiving 10 mg/kg KPT-330 every two days/week PO; Group 7: mice (10 animals) receiving 3 mg/kg/day KPT-330 PO. Treatments were started when tumor volumes reached approximately 80 mm^3^ (Day 0) and were stopped after 28 days. The following parameters were used to quantify the antitumor effects upon different treatments: (1) tumor volume measured during and at the end of experiments, (2) tumor weight measured at the end of experiment, (3) complete response (CR) defined as the disappearance of the target lesion with respect to baseline, (4) partial response (PR) defined as a reduction of greater than 50 % of tumor volume with respect to baseline, (5) stable disease (SD) defined as a reduction of less than 50 % or an increase of less than 100 % of tumor volume with respect to baseline, (6) tumor progression (TP) defined as an increase of greater than 50 % of tumor volume with respect to baseline, (7) time to progression (TTP). In vivo, combinational studies were evaluated by CalcuSyn (Biosoft). For the calculation of CI, the values of cell kill for a fixed tumor volume were considered (determined by the log cell kill (LCK)). LCK was determined as LCKZ (TKC)/(3.3KTd), where Td represents the mean control group doubling time required to reach a fixed tumor volume, expressed in days, whereas T and C are the same values as described above [54].

### Statistical analysis

Continuous variables were summarized as the mean and SD or 95 % CI for the mean. Statistical comparisons between controls and treated groups were established by carrying out the ANOVA test or by Student’s t-test for unpaired data (for two comparisons). Dichotomous variables were summarized by absolute and/or relative frequencies. For dichotomous variables, statistical comparisons between control and treated groups were established by carrying out the exact Fisher’s test. For multiple comparisons, the level of significance was corrected by multiplying the P value by the number of comparisons performed (n) according to the Bonferroni correction. Overall survival was determined by Kaplan–Meier analysis and a Gehan's generalized Wilcoxon test. When more than two survival curves were compared, the Logrank test for trend was used. This tests the probability that there is a trend in survival scores across the groups. All tests were two-sided and were determined by Monte Carlo significance. P values <0.05 were considered statistically significant. In the figures in which statistical analysis was performed, significance is indicated by an asterisk. SPSS (statistical analysis software package, IBM Corp., Armonk, NY, USA) version 10.0 and StatDirect (version. 2.3.3, StatDirect Ltd, Altrincham, Manchester, UK) were used for statistical analysis and graphical presentation.

## Results

### Expression of XPO-1 in human prostate samples

**Fig. 1:**
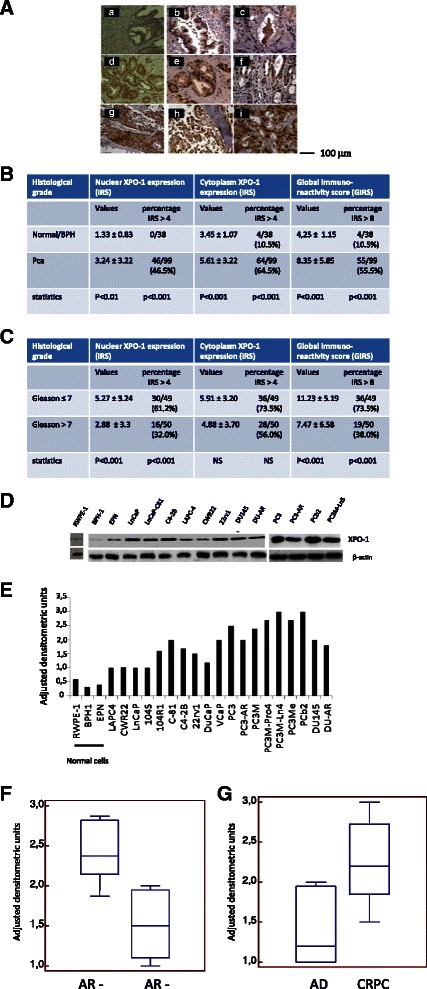
**a** CRM1 expression in normal prostate gland and BPH (**a**-**c**) and PCa of various Gleason score (**d**-**g**) and metastases (**h**, **i**). Prostate gland and BPH1 areas expressed very low levels of XPO-1/CRM1 or were negative whereas prostate cancer areas showed different CRM1 expression both in the cytoplasm and nuclei. Metastatic lesions showed very high stains mainly in the nucleus. **b**, **c** statistical evaluation of total (Global immunoreactivity score), nuclear and cytoplasm XPO-1 expression as indicated in material and methods. **d** Western blot analyses and densitometric values (arbitrary/normalized densitometric values, **e** for XPO-1 expression in some PCa and non neoplastic epithelial cells. **f** Comparison between XPO-1 in AR positive (LAPC-4, CWR22, LnCaP, LnCaP-104S, LnCaP-104R1, LnCaP-C81, C4-2B, 22rv1, DuCaP, VCaP, PC3AR and DU145AR) versus AR negative (PC3 and PC3 variants [PC3PTEN, PC3M-pro4, PC3M-Ln4, PC3Me, PCb2] and DU145) PCa cells lines. **g** Comparison between androgen-dependent (LAPC-4, CWR22, LnCaP, LnCaP-104S, DuCaP, VCaP, PC3AR and DU145AR) versus androgen independent/CRPC (LnCaP-104R1, LnCaP-C81, C4-2B, 22rv1, PC3 and PC3 variants [PC3PTEN, PC3M-pro4, PC3M-Ln4, PC3Me, PCb2] and DU145) PCa cell lines

Prostate tumors were grouped according to the Gleason score and staging. Bone metastases were also considered. In Fig. 1a, we show examples for XPO-1 expression in normal prostate gland, BPH (a-c), PCa of various Gleason score (d-g) and bone metastases (h-i). Immunoreactivity was scored as indicated in the materials and methods section. We observed that the global XPO-1 immune-reactivity scoring (GIRS) was significantly higher in PCa (8.35 ± 5.85; cases n = 99) when compared to benign hypertrophy (BPH; 5.25 ± 1.15; cases n = 38) with p < 0.001 as indicated in Fig. 1b. GIRS values were higher in Gleason scores > 7 when compared to Gleason scores ≤ 7 both when we considered GIR scores (11.23 ± 5.19 vs 7.47 ± 6.52, P < 0.001) as indicated in Fig. 1c and the percentage of patients having high GIR scores (>8) was 36/49 (73.9 %) vs 19/50 (38 %). No differences were observed in primary tumors from patients with metastatic or non metastatic tumors. Analyzing the nuclear and cytoplasm expression levels, we observed a significant increase in XPO-1 expression in the nucleus in cancer (3.24 ± 3.22) and BPH (1.33 ± 0.83) with p < 0.01 (Fig. 1b) and in Gleason scores > 7 when compared to Gleason scores ≤ 7 (5.27 ± 3.24 vs 2.88 ± 3.31, p < 0.001) and 30/49 [61.2 %] vs 16/50 [32 %] cases with high IRS (P < 0.001, Fig. 1c). The analysis performed on cytoplasm revealed differences between BPH (3.45 ± 1.07) and PCa (5.61 ± 3.22, P < 0.001, Fig. 1b) but no significant differences between values of IRS measured for Gleason scores >7 (5.91 ± 3.20) and ≤ 7 (4.88 ± 3.70, P = 0.086) or the percentage of cases with high IRS [36/49 (73.5 %) vs 28/50 (56 %)] as shown in Fig. 1c. Although the mean GIRS values showed no significant differences, the percentage of cases with high XPO-1 expression was significantly higher in primary tissues derived from metastatic compared to those observed in non metastatic cases. No statistical difference was observed in the comparisons with pathologic stage for nuclear or cytoplasm IR values. Nevertheless all metastatic lesions presented elevated GIRS and nuclear IRS for XPO-1.

### Expression of XPO-1 in human prostate cancer lines

Next, we analyzed and quantified the expression of XPO-1 in prostate cancer, normal or neoplastic prostate epithelial cells by immunoblots and by adjusted densitometry units. XPO-1 protein levels were high in prostate cancer when compared to non neoplastic prostate epithelial cells (Fig. 1d, e). Statistical analyses reveal that the XPO-1 expression levels were statistically lower in AR positive (LAPC-4, CWR22, LnCaP, LnCaP-104S, LnCaP-104R1, LnCaP-C81, C4-2B, 22rv1, DuCaP, VCaP, PC3AR and DU145AR) when compared to those observed in AR negative (PC3 and PC3 variants [PC3PTEN, PC3M-pro4, PC3M-Ln4, PC3Me, PCb2] and DU145) PCa cells lines (1.39 ± 0.38 vs 2.57 ± 0.39, P = 0.0015, Fig. 1f). The XPO-1 levels were statistically lower in androgen dependent (LAPC-4, CWR22, LnCaP, LnCaP-104S, DuCaP, VCaP, PC3AR and DU145AR) when compared to those observed in androgen independent/CRPC (LnCaP-104R1, LnCaP-C81, C4-2B, 22rv1, PC3 and PC3 variants [PC3PTEN, PC3M-pro4, PC3M-Ln4, PC3Me, PCb2] and DU145) PCa cell lines (1.41 ± 0.47 vs 2.44 ± 0.52, P = 0.0150, Fig. 1g) The comparison in PC3 cell derivatives showed higher XPO-1 levels in more aggressive/metastatic cells.

### Inhibition of XPO1 blocks growth of PCa cells

**Fig. 2:**
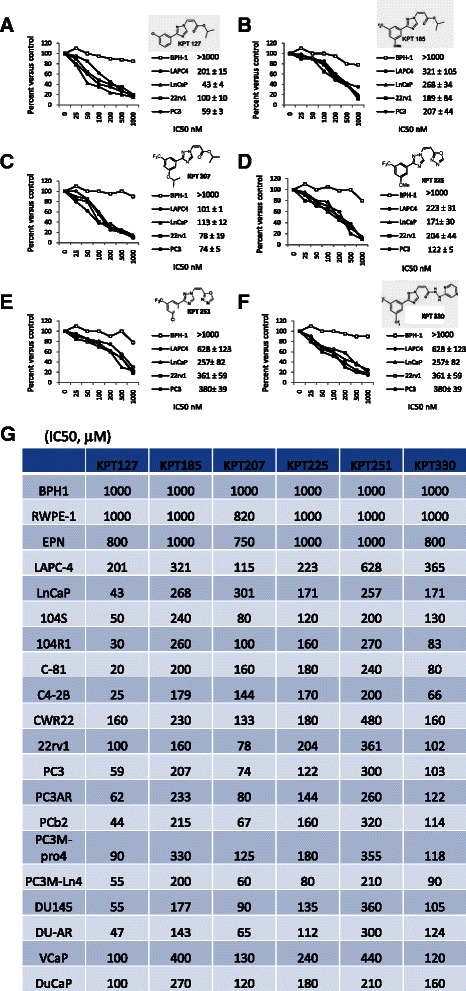
Dose-dependent effects of different KPT compounds in BPH-1, LnCaP (AR+/AD/p53wt), LAPC-4 (AR+/AD/p53mutant), 22rv1(AR+/AI/p53 wt) and PC3 (AR-/p53 null) cells treated with varying doses (0, 1, 10, 50, 100, 500 and 1000 nM) of different KPT compounds and assessed viability at 72 hours after treatment: KPT-127 (**a**), KPT-185 (**b**), KPT-207 (**c**), KPT-225 (**d**), KPT-251 (**e**) and KPT-330 (**f**). **g** IC_50_ for each compound was then calculated for the wide set of PCa cell lines

Six SINE analogs KPT-127, KPT-185, KPT-205, KPT-227, KPT251 and KPT-330 sharing a trifluoromethyl phenyl triazole scaffold [51, 52], were investigated for their growth inhibitory and apoptotic potential in our PCa cell panel. Therefore, the effects of these XPO1 inhibitors were evaluated on the growth of PCa cell lines performing dose- and time-dependent studies. To examine the dose-dependent effects of these compounds, we treated cells with varying doses of the compounds (0, 1, 10, 50, 100, 500 and 1000 nM) and assessed viability at 72 hours after treatment. The IC_50_ for each compound was then calculated. In Fig. 2a-f we show the growth curves for LnCAP, LAPC-4, 22rv1 and PC3 cells, exposure to sub-micromolar concentrations of SINE for 72 hrs. In Fig. 2g, we summarized IC_50_ values calculated in our cell systems. Most significantly, at the concentrations tested, the SINE showed no effect on growth of normal or hyperplastic prostate epithelial cells (BPH-1, RWPE-1 and EPN). We observed that all SINE compounds show good activity in PCa cell lines with IC_50_ values in the range of 43 and 700 nM. KPT-330, currently in phase I clinical trials (ClinicalTrials.gov) and KPT-127 were the most potent growth inhibitors with IC_50_ between 43 and 201 nM, whereas the least activity was observed with KPT-251 (IC_50_ values ranged between 150 and 300 nM) and KPT-185 (IC_50_ values ranged between 180 and 700 nM). Statistical analyses showed a significant correlation between efficacy of SINE compounds and XPO-1 expression with higher XPO-1 protein levels being correlated with lower IC_50_ values (and thus with higher sensitivity). The better correlation was found for KPT-127 and KPT-207 (r = −0.728 and −0.717, P < 0.005), KPT-330 (r = −0.613, P < 0.01) and KPT-251 (r = −0.485, P < 0.05). The effects of SINE compounds were higher in CRPC when compared to androgen dependent prostate cancer cell lines. Therefore the molecular analysis and the in vivo studies were made in PC3, DU145 and 22rv1 CRPC models.

### Molecular changes induced by XPO-1 inhibition

**Fig. 3:**
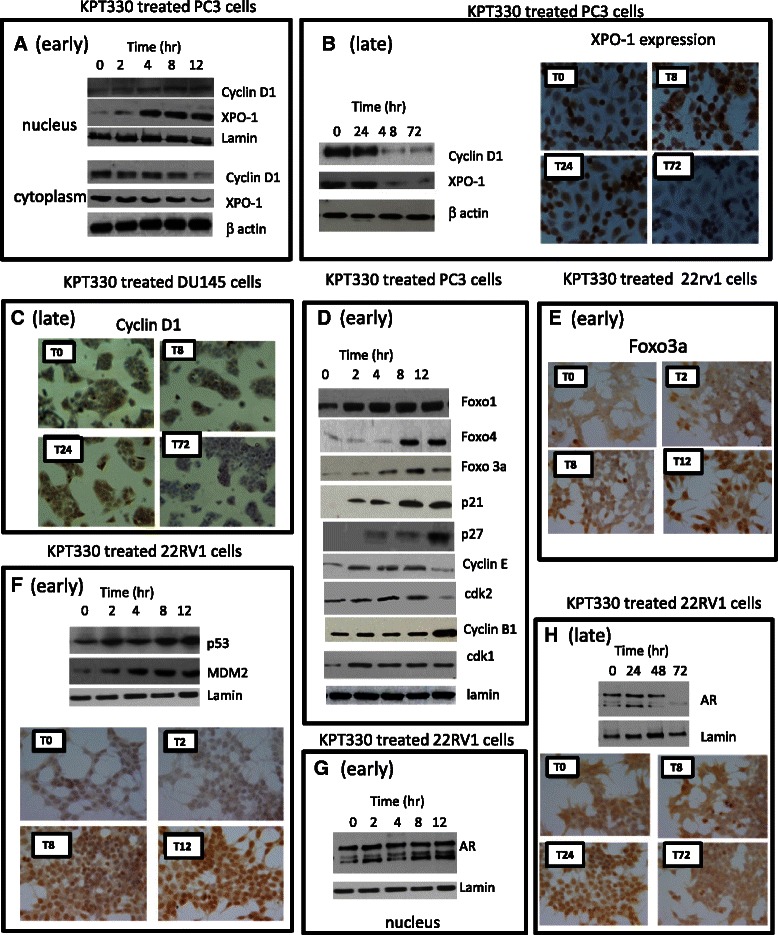
Molecular arrangements associated to CRM1 inhibition: **a**-**c** CRM1 and Cyclin D1 nuclear accumulation. **a** Western blots for the nuclear expression of cyclin D1 and CRM1 in PC3 cells treated with 100 nM KPT-330 for 4, 8, 12 and 24 hrs (early events); **b** Western blots for the cytoplasmic expression of cyclin D1 and CRM1 in PC3 cells treated with KPT-330 for 24, 48 or 72 hr and and immunocytochemical evaluation for CRM1 in the same conditions (late events) and **c** immunocytochemical evaluation for cyclin D1 expression in PC3 cells treated with 100 nM KPT-330 for 8 (T8), 24 (T24) and 72 (T72) hrs (late events). **d** Western blots for nuclear expression of Foxo proteins, p21, p27, cyclin E, cdk2, cyclin B1 and Cdk1 in PC3 cells treated with 100 nM KPT-330 for 2, 4, 8 and 12 hrs (early events). **e** Immunocytochemical evaluation for Foxo3a expression in 22rv1 cells treated with 100 nM KPT-330 for 2 (T2), 8 (T8) and 12 (T12) hrs (early events). **f** Western blots for nuclear expression of p53 and MDM2 in AR+/p53wt 22rv1 cells treated with 100 nM KPT-330 for 4, 8, 12 and 24 hrs (Early events). **g** Western blot for AR nuclear expression in 22rv1 cells treated with 100 nM KPT-330 for 2, 4, 8 and 12 hr (early events) and **h** western blot for AR nuclear expression in 22rv1 cells treated with 100 nM KPT-330 for 8 (T8), 24 (T24) and 72 (T72) hrs (late events). Nuclear expression was normalized to lamin whereas cytosolic expression was normalized to β-actin

**Fig. 4:**
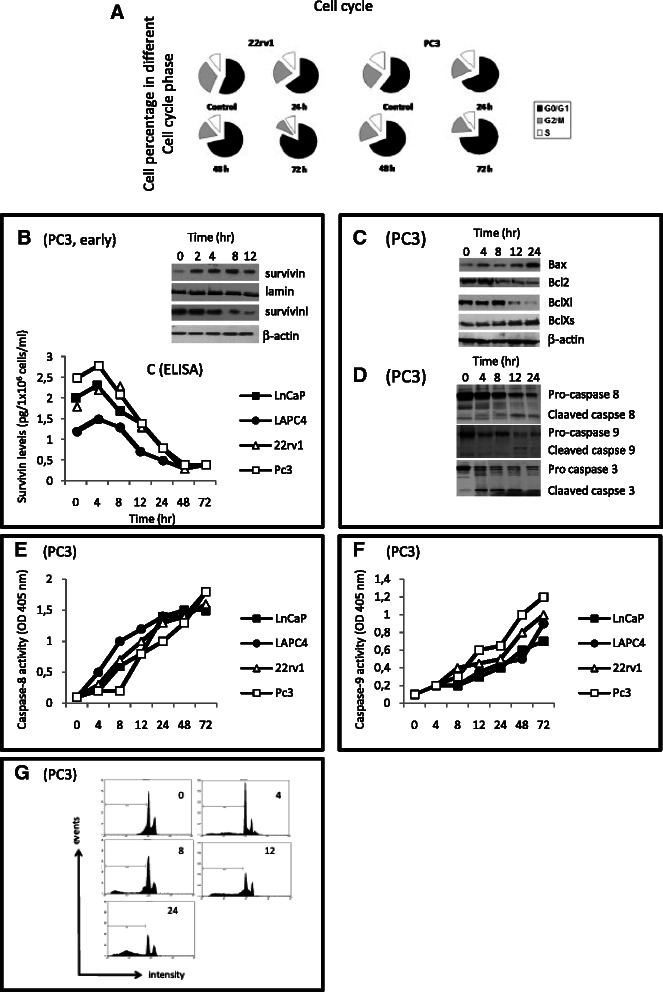
**a** Cell cycle evaluation: percentage of cells at different cell cycle phases after treatment with 100 nM KPT-330 at different times (24, 48 and 72 hrs). **b**-**g** Molecular arrangements associated to CRM1 inhibition: **b**, **c** Survivin nuclear accumulation. Western blot **b** in PC3 cells treated with 100 nM KPT-330 for 4, 8, 12 and 24 hrs. **c** ELISA determinations in PC3 cells treated with 100 nM KPT-330 for 4, 8,m 12, 24, 48 and 72; **d** western blots for protein associated with apoptosis (Bax and Bcl-2 family members) and caspases. **e** Activity of caspase 8; **f** activity of caspase 9. **g** Cytofluorimetric analysis of apoptosis

The molecular pathways that are induced by XPO-1 inhibition were evaluated by immunoblots, ELISA and immunocytochemical analyses performed in PC3, DU145 and 22rv1 as models of CRPC using 100 nM KPT-330 as reference compound. The effects were monitored in early and chronic treatments. We found that KPT-330 induced an early XPO-1 nuclear accumulation. In parallel, cyclin D1 nuclear expression was increased whereas cytosolic levels were strongly reduced. However, prolonged treatments with KPT-330 dramatically reduced XPO-1 protein levels. In Fig. 3a, we show representative western blots for nuclear and cytoplasm expression of cyclin D1 and XPO-1 in PC3 cell extracts after 2–12 hr of treatment with KPT-330. In Fig. 3b, we demonstrate that total XPO-1 levels were also reduced after prolonged treatment of PC3 cells with KPT-330. Western blot and immune-cytochemistry showed similar results. In addition, prolonged treatment with KPT-330 determined a reduction of cyclin D1 protein expression in DU145 cell lines (Fig. 3c). Western analysis demonstrated a nuclear accumulation of FOXO, p21, p27, cyclin B1 as shown in Fig. 3d for PC3 cells. Increased nuclear expression of FOXO3a is visible also by IHC (Fig. 3e) in 22rv1 cells treated with KPT-330. In addition, we found that KPT-330 treatment increased nuclear expression of p53 and MDM2 (Fig. 3f) and AR (Fig. 3g) in AR+/p53wt 22rv1 cells. Also in this case, prolonged exposure to KPT-330 significantly reduced the total expression of AR, especially in the truncated androgen insensitive forms. Indeed, it has been demonstrated that AR expression is increased in CRPC and XPO-1 activity is necessary for a completely functionally active AR-mediated pathway. Therefore, the inhibition of XPO-1-mediated export machinery may enhance AR signaling (manuscript in preparation). In addition, the disrupted cyclin B1-XPO-1 interactions leading to a marked nuclear accumulation of cyclin B1 is necessary for cyclin B1-dependent apoptosis In Fig. 3h, we demonstrate that proteasome inhibition was able to block the degradation of XPO-1, Cyclin D1 and survivin reducing the loss of these proteins induced by KPT-330 treatment. Although proteasome inhibitors are considered for treatment of solid tumors, these compounds could reduce the effectiveness of selinexor, requiring further investigation. These molecular changes were associated with accumulation of cells in the G1 cell cycle phase with loss of cells in the S and G2/M phase (Fig. 4a).

Western blots and ELISAs revealed a reduced nuclear export of survivin in PCa cell lines after KPT-330 administration. In Fig. 4b and c, we show the early reduction of cytoplasmic survivin and increase of nuclear accumulation in PC3 cells treated for different times with 100 nM KPT-330. A decrease in cytosolic an increase in nuclear survivin protein expression was observed as early as 4 hours after treatment. Survivin continued to accumulate within the nucleus until 12 hours after treatment, after which time it decreased dramatically in all cellular compartments.

Immunoblot analysis also showed changes in the Bcl-2 family proteins: Bax, Bcl-2, Bcl-Xl and Bcl-Xs, suggesting a role in apoptosis (Fig. 4d). This was associated with an increased caspases-3 dependent PARP cleavage (Fig. 4e-g). FACS and Histone/DNA ELISA analyses indicated an increased apoptosis. In Fig. 4h we show the dose and time-dependent apoptosis measured in aggressive PC3, 22rv1, DU145 and C4-2B cell lines.

### XPO-1 inhibition slows prostate tumor growth in vivo

**Fig. 5:**
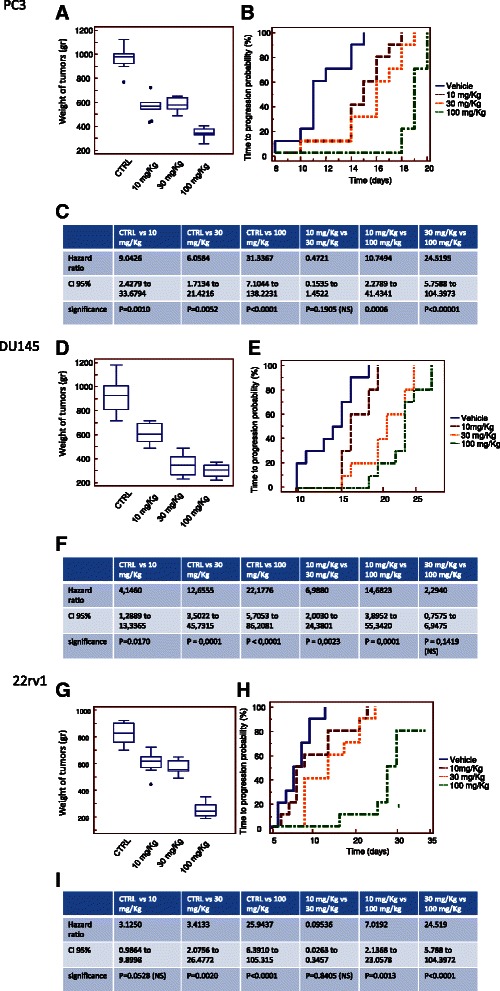
KPT-251 inhibits prostate tumor growth in vivo. Once tumors were established (80–100 mm3 in size), mice were divided into four different groups (10 mice/group) and treated with varying doses of KPT-251 (10, 30, 100 mg/kg Mondays, Wednesdays and Fridays PO). Tumor volumes were calculated as indicated in MM and followed for 35 days. Group means were calculated and are shown with error bars representing SD for each group. **a** Tumor weight of PC3 xenografts treated or not with KPT-251. **b** Time to Progression (TTP) defined as the time (days) necessary to double the tumor volume for each tumor, with Kaplan Meyer curves and calculated in DU145 xenografts. **c** Hazard ratio values. **d** Determination of tumor weight in DU145 xenografts treated or not with KPT-251. **e** Time to Progression (TTP) **f** Hazard ratio values. **g** Determination of tumor weight in 22rv1 xenografts treated or not with KPT251. **h** Time to Progression (TTP) calculated in 22rv1 xenografts. **i** Hazard ratio values

**Fig. 6:**
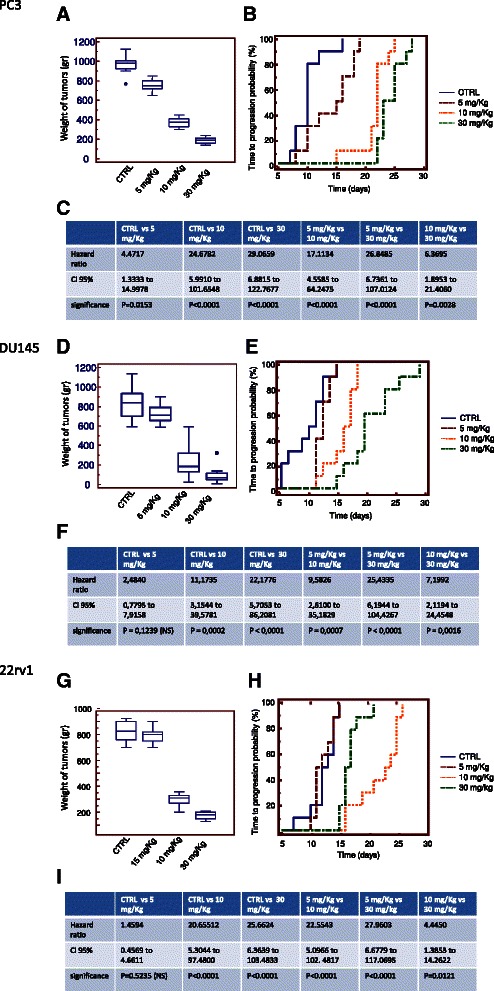
KPT-330 inhibits prostate tumor growth in vivo. Once tumors were established (80–100 mm3 in size), mice were divided into four different groups (10 mice/group) and treated with varying doses of KPT-251 (5, 10, 20 mg/Kg Mondays, Wednesdays and Fridays PO). Tumor volumes were calculated as indicated in MM and follow for 35 days. Group means were calculated and are shown with error bars representing SD for each group. **a** Tumor weight of PC3 xenografts treated or not with KPT-330. **b** Time to Progression (TTP) calculated in PC3 xenografts. **c** Hazard ratio values. **d** Determination of tumor weight in DU145 xenografts treated or not with KPT330. **e** Time to Progression (TTP) calculated in DU145 xenografts. **f** Hazard ratio values. **g** Determination of tumor weight in 22rv1 xenografts treated or not with KPT-330. **h** Time to Progression (TTP) calculated in 22rv1 xenografts. **i** Hazard ratio values

To determine the effects of XPO-1 inhibition on prostate cancer growth in vivo, we used two aggressive CRPC, PC3, DU145 and 22rv1, cell lines engrafted in male nude mice. Once tumors were established (80–100 mm3 in size), mice were divided into seven different groups (10 mice/group) and treated with varying doses of KPT-251 (10, 30, 100 mg/kg PO QoDx3) and KPT-330 (5, 10, 20 mg/kg PO QoDx3). Tumors were measured for 35 days. The effects of KPT-251 on tumor growth is shown in Fig. 5 and KPT-330 in Fig. 6. The mean tumor weight was calculated for each treatment group. Figure 5 shows that KPT-251 was able to reduce tumor weight in a dose-dependent manner in PC3 (a-c), DU145 (d-f) 22rv1 (g-i) xenografts. Statistically significant differences were observed between the vehicle control groups and the groups treated with KPT-251 at 10 mg/kg on Mondays, Wednesdays, and Fridays (P < 0.0001 in 22rv1 and DU145and P = 0.023, in PC3 xenografts), KPT-251 at 30 mg/kg on Mondays, Wednesdays, and Fridays (P < 0.0001 in 22rv1 and DU145 and P = 0.002 in PC3 xenografts), and KPT-251 at 100 mg/kg on Mondays, Wednesdays and Fridays (P < 0.0001 for all xenografts). ANOVA analysis showed similar statistical significance (p < 0.0001) in all cell models. In order to reduce the probability of an error due to the tumor volume at the start of drug administration we compared the Time to Progression (TTP) defined as the time (days) necessary to double the tumor volume for each tumor. The comparison of TTP (Fig. 5b, e and h) shows a better behavior of KPT-251 in 22rv1 and DU145 when compared to the PC3 xenograft in terms of hazard ratio values (Fig. 5c, f and i).

**Fig. 7:**
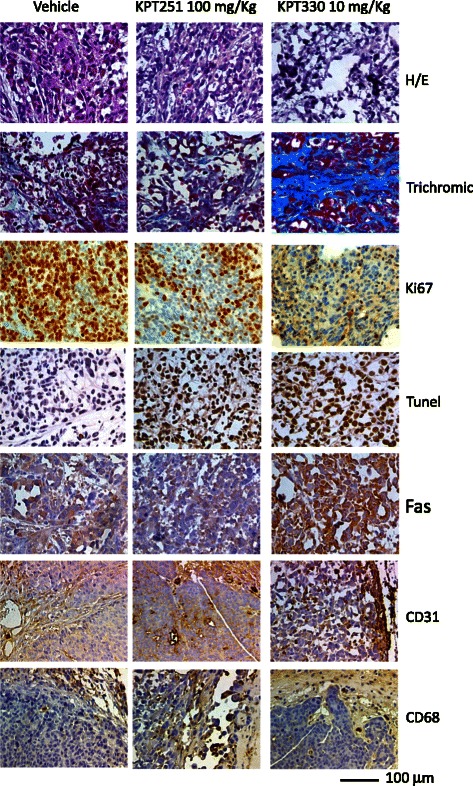
Histochemical and immunohistochemical analyses. Hematoxylin/Eosin and Trichromic staining was shown in PC3 xenografts treated with KPT-251 (100 mg/kg) and KPT-330 (10 mg/kg). In this panel was also shown the expression of K67, tunnel, CD31, Fas and CD68 staining, depicting respectively proliferation, apoptosis, vessel formation, cell death receptor and monocyte/macrophage presence

Figure 6 show that KPT-330 was able to reduce tumor weight in a dose-dependent manner in PC3 (a-c), DU145 (d-f) 22rv1 (g-i) xenografts. Statistically significant differences were observed between the vehicle control groups and the groups treated with KPT-330 at 5 mg/kg on Mondays, Wednesdays, and Fridays but not for DU145 cells (P = 0.014 and P = 0.003, in 22rv1 and PC3 xenografts, respectively), KPT-330 at 10 mg/kg on Mondays, Wednesdays, and Fridays (P < 0.001 in all xenografts), KPT-330 at 30 mg/kg on Mondays, Wednesdays and Fridays (P < 0.001 in all xenografts). Also in this case, tumor growth reduction by KPT-330 treatment had a similar behavior in all cell models and the comparison of TTP shows improved hazard ratio values of KPT-330 in 22rv1 and DU145 when compared to PC3 xenograft (Fig. 6c, d). 22rv1 and DU145 cells are more sensitive to XPO-1 block when compared to PC3 cells. KPT-330 had better antitumor effects at similar dosage compared to KPT-251. Histochemical and immunohistochemical anlaysis of tumors treated with KPT-251 (100 mg/kg) or KPT-330 (10 mg/kg showed evidence of cell cycle arrest and apoptosis after treatment. Sectioned tumors following 42 days of treatment were stained with hematoxylin/eosin, trichromic stain, Ki-67, TUNEL, CD31, CD68 and Fas. The results show decreased cell proliferation, seen by the reduction in the number of Ki-67–positive cells and increased apoptosis as seen by increase in TUNEL-positive cells. The reduction in tumor cells can be seen by the H&E stain as well as with increased fibrosis, as demonstrated by increased trichromic stain (Fig. 7). The induction of Fas suggests that SINE may induce apoptosis through the activation of the Fas receptors. Increased staining of CD31 indicates the exposure of blood vessels in treated shrinking tumors and the increase in CD68 indicated accumulation of monocytes and macrophages in the areas of the treated tumors (Fig. 7).

**Fig. 8:**
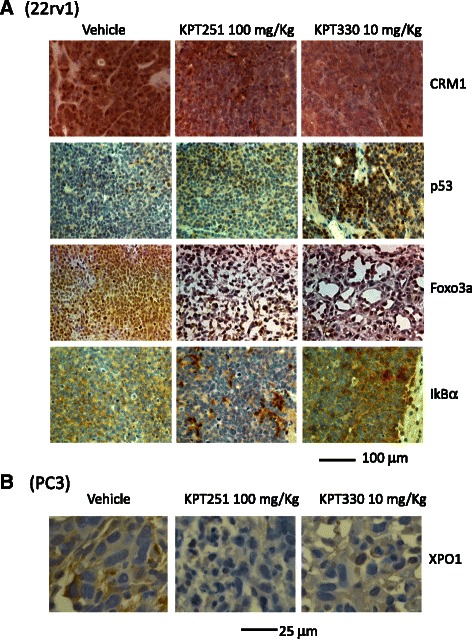
**a** Immunohistochemical evaluation of XPO-1, p53, Foxo3a and IkBa performed in 22rv1 xenografts treated with or without KPT-251 or KPT-330. In this fig. is shown XPO-1, p53, Foxo3a and IkBa expression to demonstrate the changes in nuclear expression and protein level induced by KPT-251 (100 mg/kg) and KPT-330 (10 mg/kg) treatments. **b** Changes in the expression of XPO-1 in PC3 xenografts treated with or without KPT-251 or KPT-330

Staining of tumor biopsies for XPO1 and selected protein cargos shows a reduction in total XPO1 protein levels in treated tumors and increased nuclear localization of p53, FOXO3 and IκBα (Fig. 8).

## Discussion

Multiple tumor suppressor proteins are mislocalized in cancer cells by overexpressed XPO-1 [13–23]. Here, we demonstrate that nuclear and cytoplasmic expression of XPO-1 is elevated in prostate tumors compared to normal and hyperplastic tissue. In addition, XPO-1 IR is higher in Gleason score > 7 and metastases, which could be associated to its function. In vitro aggressive/metastatic PCa cells show higher levels of XPO-1 when compared to less aggressive cells, and castration resistant cells show higher XPO-1 levels when compared to androgen dependent cells.

Our results show that SINE compounds, which inhibit XPO1 activity, have anticancer effects in in vitro and in vivo models of human cancer. In this report, we also demonstrate that inhibition of XPO-1 is a potential target for the treatment of aggressive/castration resistant PCa. We tested a battery of SINE compounds in PCa cell lines and KPT-330 and KPT-251 were tested in vivo using two models of aggressive PCa. The results of our study demonstrate that SINE compounds are potent anticancer agents in these models. The clinical candidate, KPT-330, significantly reduced tumor cell growth to approximately one-third the volume of tumors observed in docetaxel–treated animals (internal control, data not shown). Reduction in tumor growth was dose-dependent and associated with inhibition of cellular proliferation and activation of apoptosis, which correlated with our in vitro findings showing PARP and caspase-3 cleavage. SINE compounds are potent therapeutic tools to treat aggressive/castration resistant PCa cells. This appears to be due to the modulation of a multiple signaling pathways.

It has been demonstrated that cyclin D1 overexpression increases the progression of PCa [55] and cyclin D1 knockdown reduce cell proliferation and increases sensitivity to radiotherapy and chemotherapy [56]. Cyclin D1 nuclear overexpression induces differentiated phenotype in B-cell lymphoma in transgenic mice [57] and drives the oncogenic transformation of murine fibroblasts [58]. Cyclin D1 is sequestered in the cytoplasm of mammalian cancer cells [59], where the enforced nuclear localization of cyclin D1 induces apoptosis. Thus, the subcellular localization of cyclin D1 may play a role in cell survival. The competing processes of nuclear import and export induce cyclin D1 localization [60]. Here, we observed that SINE compounds inhibit cell cycle progression, increase early nuclear expression and reduce late cyclin D1 expression PCa cells. Cyclin D1 overexpression and abnormalities in cell-cycle inhibitory genes p21WAF1, p16INK4a, and p27KIP1 have been reported in PCa [61]. P21WAF1 mainly localizes to the cytoplasm in many tumor cells, and cytoplasmic P21WAF1 is anti-apoptotic. P21WAF1 prevents cell cycle progression at the G1 phase. KPT-330 and KPT-251 reduce the export of P21WAF1 from the nucleus and increase nuclear expression of this protein. This was associated with reduced Ki67 and increased tunnel staining. Our data is in agreement with those observed by Van der Watt et al., [62] which found that XPO-1 inhibition significantly reduces cell proliferation and increases apoptosis and P21 nuclear localization.

Exogenous and endogenous stress can activate ATM, a DNA damage sensor that activates the tumor suppressor p53, which, in turn, inhibits cell cycle progression and activates DNA repair mechanisms. p53 is often inactivated in PCa due to its deletion or mutation [63]. However, p53 activity can be also regulated by its subcellular localization. p53 mis-localization arising from an aberrant import mechanism, hyperactive export, or sequestration with a cytoplasmic factor has been observed in several cancers. Normally, the nuclear-cytoplasmic transportation of p53 is tightly regulated. Here, we demonstrated that KPT-330 or KPT-251 are able to block transport of p53 from the nucleus, leading to its activation, cell cycle arrest, and apoptosis in p53 wt 22rv1 cells as previously demonstrated with leptomycin B [24]. Furthermore, down regulation of both p21/Cip1 and p27/Kip1 produces a more aggressive PCa phenotype [62]. The nuclear localization of P27KIP1 enables this regulatory function. However, the nuclear export of P27KIP1 is mediated by the XPO-1 export receptor. Hence, XPO-1 inhibition may restore the negative regulatory function of P27KIP1 in prostate cell cycle progression.

The constitutive activation of the PI3K pathway is key to PCa cell survival [9, 10] due to growth factor activation or phosphatase and tensin homolog (PTEN) loss. In normal cells, PTEN, the cellular PI3K antagonist, can inhibit PI3K activation, resulting in the nuclear localization of Forkhead Box O (FOXO) transcription factors, involved in multiple signaling pathways and having tumor-suppressor functions. In PCa deregulation of oncogenic kinases, including Akt, extra-signal-regulated kinase, or IκB kinase, is frequently observed, which may potentially inactivate FOXO activity. FOXO3a is, indeed, in a constant inactive state due to its cytoplasmic localization. In the nucleus, FOXO can activate the transcription of genes that promote cell cycle arrest and apoptosis [64]. Thus, localizing FOXO to the nucleus is beneficial to controlling cell survival. The constitutive activation of PI3K constitutively activates protein kinase B (AKT) which phosphorylates FOXO transcription factors at multiple sites, thereby preventing FOXO-DNA binding and transcriptional activities, as well as promoting the XPO-1-dependent export of FOXO from the nucleus. We observed that KPT-251 and KPT-330 significantly induce nuclear localization of FOXO3a. FOXO re-localization to the nucleus, where it becomes active, is a promising method of controlling cell proliferation.

The transcriptional activator nuclear factor-κB (NF-κB) has been implicated in tumorigenesis and resistance therapy. Advanced/aggressive PCa cells express constitutively activated NF-κB [65]. Here we observed that IkBα expression is increased in the nuclei of cells when 22rv1- or PC3-bearing mice were treated with KPT-330. Therefore, the perturbation of the XPO-1-dependent nuclear export of IκBα may attenuate constitutively activated NF-κB and cause immediate apoptosis in PCa cells.

The Fas/FasL system is significant in tumorigenesis and a previous investigation has indicated that the impairment of the Fas/FasL system in cancer cells may lead to apoptosis resistance and contribute to tumor progression [66]. We observed that KPT-330 and KPT-251 induced the expression of FAS in vitro and in vivo. This increase could be responsible for the elevation of caspase 8 activity observed in vitro. Therefore FAS/FASL system could be activated and FASL produced in the tumor microenvironment by pro-inflammatory and resident stromal cells could participate to the XPO-1 mediated cell death. We also observed increased fibrosis and necrosis associated to increased percentage of CD68 monocytes/macrophages. Taken together these observations could further increase local FAS/FASL activity. In addition, it is previously shown that FOXO3A when active (nuclear localization) induces the expression of the FAS ligand protein [67].

High levels of survivin expression are independent risk factors for poor prognosis in cancer [68]. Cytoplasmic survivin has been shown to be particularly high in prostate tumors and to be an independent predictor of poor prognosis, whereas nuclear survivin has been a favorable factor [68, 69] in some studies. These clinical results support the notion that nuclear survivin is suppressive for tumor growth, and further that targeting the cytoplasmic, antiapoptotic fraction of survivin would be an ideal therapeutic avenue. As survivin requires XPO-1/XPO1-RanGTP for its nuclear exit, inhibiting the activity of this complex could directly address this therapeutic need by increasing the tumor-suppressive nuclear survivin and reducing the antiapoptotic cytoplasmic survivin. Our results suggest that survivin is an essential component of the downstream signaling pathway of XPO-1 inhibition in prostate cancer cells, intriguingly an increase in the total concentration of survivin can interfere with these drugs' antitumor effects. Treatments with KPT-330 or KPT-251 led to an initial decrease in cytoplasmic survivin protein levels with a corresponding increase in its nuclear expression. This result was transient however, as the major effect was to deplete total survivin levels. Interestingly, other reports have shown a decrease in total survivin following treatment with LMB [28], and more recently it was reported that the NF-κB–dependent survival factor, Mcl1, is depleted in response to KPT-185 [38]. Taken together, these results provided unequivocal proof of the potential SINEs as new class of anticancer drugs. Our results suggest that SINEs initially promote survivin nuclear localization, but at later time points leads to a reduction in its protein levels correlating with the timing of cellular antitumor effects of these compounds and supporting a hypothesis that XPO-1 inhibition leads to a loss of survivin levels which tend to lead to inhibition of tumor cell growth and enhanced tumor cell apoptosis.

## Conclusions

We demonstrated that the SINE KPT-330, recently demonstrated to inhibit bone metastases formation in PCa [43] and currently in phase I clinical studies in humans (NCT01607905), is an interesting therapeutic target for advanced/castration resistant prostate tumors.
