# Erratum to: KPT-330, a potent and selective exportin-1 (XPO-1) inhibitor, shows antitumor effects modulating the expression of cyclin D1 and survivin in prostate cancer models

**DOI:** 10.1186/s12885-015-2046-7

**Published:** 2016-01-11

**Authors:** Giovanni Luca Gravina, Andrea Mancini, Patrizia Sanita, Flora Vitale, Francesco Marampon, Luca Ventura, Yosef Landesman, Dilara McCauley, Michael Kauffman, Sharon Shacham, Claudio Festuccia

**Affiliations:** Department of Biotechnological and Applied Clinical Sciences, Laboratory of Radiobiology, University of L’Aquila, L’Aquila, Italy; Pathology Division, San, Salvatore Hospital, L’Aquila, Italy; Karyopharm Therapeutics, Newton, MA USA

Unfortunately, the original version of this article [1] contained a mistake. The word ‘survivin’ in the title was incorrectly included as ‘surviving’. The title has been corrected above and in the original article.
